# Annals of Surgery

**Published:** 1891-12

**Authors:** 


ANNALS OF SURGERY. A Monthly Review of Surgical Science and Practice, edited by L. S. Pilcher, A. M., M. D., of Brooklyn, and C. B. Keetley, F. R. C. S., of London, England. Price, $5.00 per year, in advance.

The November number of this excellent journal is before us. The principal article is an interesting description of Cysts of the Urachus, illustrated with three drawings. We have repeatedly called attention to this journal as a most desirable periodical for every physician who wishes to know the latest advances in surgery. W. M. J.

